# Translation of the geriatric emergency department intervention into other emergency departments: a post implementation evaluation of outcomes for older adults

**DOI:** 10.1186/s12877-022-02999-4

**Published:** 2022-04-07

**Authors:** Elizabeth Marsden, Alison Craswell, Andrea Taylor, Adrian Barnett, Pan-Kar Wong, Marianne Wallis

**Affiliations:** 1 Sunshine Coast Hospital and Health Service, Sunshine Coast University Hospital, 4 Doherty Street, Birtinya, QLD 4575 Australia; 2 University of the Sunshine Coast, Sunshine Coast Health Institute, 4 Doherty Street, Birtinya, QLD Australia; 3 Queensland University of Technology, 60 Musk Avenue, Kelvin Grove, Brisbane, Qld 4059 Australia; 4 Gold Coast Hospital and Health Service, Gold Coast University Hospital, 1 Hospital Blvd, Gold Coast, Qld 4215 Australia; 5 Southern Cross University, Southern Cross Drive, Bilinga, QLD 4225 Australia

**Keywords:** Geriatric, Emergency medical services, Emergency nursing, Knowledge translation, Implementation, Homes for the aged, Delivery of healthcare, Aged care

## Abstract

**Background:**

Increasing numbers of older adults with complex health deficits presenting to emergency departments has prompted the development of innovative models of care. One such model designed to reduce poor outcomes associated with acute healthcare, is the Geriatric Emergency Department Intervention. This intervention is a nurse-led, physician-championed, Emergency Department intervention that improves the health outcomes for frail older adults in the emergency department.

**Methods:**

This quantitative cohort study aimed to evaluate the healthcare outcomes and costs associated with the implementation of the Geriatric Emergency Department Intervention (GEDI) for adults aged 70 years and over at two hospital sites that implemented the model using the integrated-Promoting Action on Research Implementation in Health Services (i-PARHIS) framework. Hospital A was large teaching hospital located in the tropical north of Australia. Hospital B was a medium sized teaching hospital near Brisbane, Queensland Australia.

The effect of the intervention was examined in two ways. Outcomes were compared between: 1) all patients in the pre- and post- implementation periods, and 2) patients seen or not seen by the Geriatric Emergency Department Intervention team in the post-implementation period. The outcomes measured were disposition (discharged home, admitted); emergency department length of stay; hospital length of stay; all cause in-hospital mortality within 28 days; time to re-presentation up to 28 days post-discharge; emergency department and in-hospital costs.

Survival analyses were used for the primary and secondary outcome variables and a Cox survival model was used to estimate the associations between variables and outcomes. Multiple regression models were used to examine other secondary outcomes whilst controlling for a range of confounders.

**Results:**

The Geriatric Emergency Department Intervention was successfully translated into two different emergency departments. Both demonstrated an increased likelihood of discharge, decreased emergency department length of stay, decreased hospital costs for those who were admitted, with an associated reduction in risk of mortality, for adults aged 70 years and over.

**Conclusions:**

The Geriatric Emergency Department Intervention was successfully translated into new sites that adapted the model design. Improvement in healthcare outcomes for older adults presenting to the emergency department was demonstrated, although this was more subtle than in the original model setting.

**Supplementary Information:**

The online version contains supplementary material available at 10.1186/s12877-022-02999-4.

## Background

Health systems are experiencing increasing pressure from ageing populations as more patients, with multiple comorbidities, present to emergency departments (ED) with acute illnesses or exacerbations of their underlying chronic health conditions. Previous research suggests that a longer time in the ED is associated with increases in unnecessary investigations, admission rates, prolonged hospitalisation, and higher in-hospital mortality, for this older cohort [1, 2]. This evidence has stimulated the development of a range of models of enhanced care for older adults in the ED (c.f.^3^, ^4^, ^5^). The evidence from these models of care, indicates that a targeted comprehensive geriatric assessment, undertaken with older adults, results in improved short- and long-term health outcomes [6]. One of these models is the Geriatric Emergency Department Intervention (GEDI), which was successfully developed and trialled in a regional Queensland ED and reduced ED length of stay, hospital admission, hospital length of stay and cost, without increasing mortality or same cause re-presentation with increases sustained over time [5, 7, 8].

EDs provide a rich and complex landscape in which to undertake innovation research, such as with the GEDI model [9]. Innovation research studies provide clinicians with new and exciting ways to potentially improve the efficiency and effectiveness of ED healthcare delivery [10]. However, despite sound evidence, the dissemination of innovations into other EDs is often slow or non-existent [10]. Diffusion and implementation sciences have developed, and these methodologies enable organisational change scholars to assist clinicians in the adoption and long-term sustainability of evidenced based innovations [11]. Currently there are over 100 reported theories spanning several disciplines that are being used by implementation scientists for the planning, evaluation and dissemination of project outcomes [12]. However, the most cited theories include Diffusion of Innovations [13], integrated-Promoting Action on Research Implementation in Health Services (iPARIHS) [14], Theoretical Domains Framework [15] and the Consolidated Framework for Implementation Research [16].

In this study, the i-PARIHS framework [14] was used to assist the implementation processes at two sites. This framework was chosen as the initial version (PARIHS) was developed after experience working with nursing clinicians to assist them to improve quality of care though standards, introduce audit and quality improvement and change patient services [17]. This article presents the results of a study that evaluated the effectiveness of the GEDI model in the two Queensland EDs following structured implementation.

## Methods

### Aims

An implementation science project was undertaken which focused on the implementation the GEDI model [5] into other hospital sites. Both quantitative and qualitative data were collected. The overall aim was to evaluate the effectiveness of the implementation of the GEDI model of care into two EDs. This article presents the quantitative results from the analysis of the effect of the GEDI implementation on healthcare outcomes and costs. Another article reporting the results of the qualitative study has been submitted for publication elsewhere.

In this quantitative study, the objectives were to compare the health system outcomes of: disposition, ED and Hospital length of stay (LoS), ED representation and hospital readmission, all-cause mortality and costs. The analyses focused on comparing patients aged 70 years and older, who a) presented before and after the introduction of the GEDI model and b) were seen or not seen by GEDI staff.

### Settings

Two Hospital and Health Services EDs in Queensland were chosen as the GEDI implementation sites. The senior ED clinicians and managers at these hospitals had shown early interest in implementing the GEDI model, given the initial trial results, and the supportive funding from the Healthcare Improvement Unit of Queensland Health (the state health department) that was available to support such implementation [5, 18]. Hospital A was large teaching hospital located in the tropical north of Australia. Hospital B was a medium sized teaching hospital near Brisbane, Queensland Australia.

### Description of site interventions

The intervention has been previously described and is outlined within an Implementation Toolkit and prior publications [5, 18]. Briefly, the GEDI model consists of advanced practice nurses, with at least five years experience in geriatrics, who operate an embedded subspecialty team that assists the primary ED nurses and physicians, focused on the care of older adults aged 70 years and older. The GEDI nurses are employed in the ED and worked Monday to Friday 07.00 to 17.30 h and 07.00 to 15.30 h on weekends. An embedded ED physician champion assists in ensuring acceptance and permanence of the model. During this study, GEDI nurses used the interRAI ED Screener [19] to screen all patients aged 70 years and older after presentation to the ED to prioritise patients to be seen. This tool provides a rapid measure of physical and cognitive dependence as a proxy measure for frailty. The priorities were: all patients presenting from residential aged care facilities, high functioning older adults who might have suffered incapacitating fractures (such as neck of femur), all patients with interRAI scores of 5–6, those identified as carers for a highly dependent person, and lastly, those with interRAI scores of 3–4. These patients were considered most at risk of experiencing poor healthcare outcomes, such as re-presentation to the ED, hospital admission and longer ED length of stay [20].

In the implementation process, however, both sites modified the model. This was not discouraged as an intervention should be allowed to adapt to local environments to ensure increased likelihood of success [21]. Hospital A’s ED physician champion left approximately three months into the implementation and was not replaced. Some aspects of this role were then undertaken by a supportive geriatrician but this role was not based in the ED. Hospital A had a specialist gerontology nurse leading the service and internally employed ED nurses, with an interest in older adults, rather than employing specialist gerontology nurses as GEDI nurses. Hospital A also introduced a pharmacist to the GEDI team. Hospital B had a specialist gerontology nurse leading the service, non-ED nurses with gerontology expertise and an emergency physician who operated as a change agent but did not continue as a champion for the service. In Hospital B, a geriatrician and a physiotherapist were included in the team. Several GEDI nurses were employed over the implementation period, none staying in the role for the full 12 months.

### Approach to Implementation

The implementation of the GEDI model used the iPARIHS approach to facilitation [14]. The implementation process was managed by two types of facilitators, external and internal. The external facilitators consisted of the ED physician and senior GEDI nurse who had been involved in the initial GEDI trial. At each implementation site there were two internal facilitators who acted as local champions. The GEDI Toolkit [18] was used as a resource, and site visits by internal facilitators to the initial trial site were made. The internal facilitators and the senior GEDI nurse would have regular telephone and videoconference meetings. Further details of the planned implementation activities and how reality deviated from the plans is reported in the qualitative findings from the study submitted elsewhere.

### Participants and data collection

De-identified data for all patients aged 70 years and older, who presented to the study EDs from Jan 2017 to Dec 2018 for Hospital A and from July 2016 to September 2018 for Hospital B were retrieved from the electronic medical databases of the participating hospital and health services. Data extraction was undertaken by health service data managers linking ED, inpatient and financial data. Pre-GEDI is the period prior to the introduction of the GEDI model into the departments. For Hospital B, the period between the 30 June and 1 September 2017 was not included in the analysis as some GEDI staff, but not all, were working during this time. Post-GEDI is the period after GEDI was fully established in the department. The dates for pre- and post-periods and numbers of presentations are in Table 1.

**Table 1:** Hospital A and B Pre- and Post-GEDI presentations

Hospital	Numbers of patients aged 70 + presenting pre-GEDI	Numbers of patients aged 70 + presenting post-GEDI
Hospital A	9,391	9,349
Hospital B	8,933	9,899

*GEDI* Geriatric Emergency Department Intervention

Independent variables that were used to describe the sample and to build multivariable models to compare outcomes included demographics such as age, sex and Indigenous status, the date and time of presentation and the clinical diagnosis determined by the reason for presentation as an ICD-10 code, assigned in the ED. The ICD-10 code was mapped to 25 major diagnostic categories established by Clinical Excellence, Queensland Health [22]. Other independent variables included: Intervention group (explained below) arrival by ambulance and the Australasian Triage Score (ATS) [23] grouped into three categories (ATS 1&2, ATS 3 and ATS 4&5). The primary outcome variable was disposition (admitted or discharged). Secondary outcome variables were: All cause in-hospital mortality; ED LoS (in minutes); Hospital LoS (in days); ED Representation within 28 days for any reason; Readmission within 28 days for any reason; Cost of ED presentation; Cost of hospital admission. Hospital costing data were provided by the hospitals’ financial systems in a system where funding is DRG (diagnostic related group) based and variations from DRG are individually costed at patient level post discharge.

### Statistical methods

A statistician, independent to the study team (AB) provided statistical summaries and analysis. Descriptive statistics were used to describe the patients’ characteristics in the two time periods.

GEDI was examined in two ways:

- Pre- and post-GEDI periods: comparing all patients in the post-period with all patients in the pre-period (assessing overall impact of GEDI implementation).
- Seen by GEDI: comparing patients in the post-GEDI period that were either seen or not seen by a GEDI clinician (assessing the impact of the GEDI model).

We used survival analysis for the primary outcome given the variables were times and subject to censoring from competing risks. Cumulative probability curves were plotted to examine the competing risks of admission and discharge after ED presentation and graphically show any difference due to the intervention. We used a competing risk survival approach to model the risks of admission and discharge, and how these risks depended on the independent variables. A Cox survival model was used to estimate the associations between variables and outcomes. We also used survival analysis for the secondary outcomes involving times, that is re-admission and re-presentation within 28 days, and censored patients who had not returned at 28 days. Results are presenting using means and 95% confidence intervals.

Multiple regression models were used to examine other secondary outcomes including all cause in hospital mortality, ED and hospital LoS and costs. In these models the reference categories for independent variables were:

- Mode of Arrival = Other
- Triage priority = Less urgent/Non-urgent
- Gender = Female
- Post-GEDI period = Pre-GEDI

Multiple regression was used to estimate how the GEDI model changed the key outcomes whilst controlling for a range of confounders based on expert knowledge of the system, which were age, gender, ATS, model of arrival, and major diagnostic group codes of trauma and cardiac. Multiple linear regression was used to examine differences in ED and hospital costs. Multiple logistic regression modelling was used to estimate the risk of in-hospital death. For the logistic model we present the results as prevalence ratios rather than odds ratios because odds ratios are often misinterpreted as prevalence ratios. We also log-transformed (base e) variables that had a strong positive skew in the plots and regression models. This was to give a better visual indication of any difference in the plots, and clearer picture of any differences in the regression models.

Checks of the residuals for all models were made to look for outliers, skew and bimodality. These checks were adequate and included the cost models – which are often positively skewed. Hospital LoS had a strong positive skew and so was log-transformed (base e) prior to modelling, and the estimates back-transformed to give the results on the original scale of days.

## Results

A total of 18,740 records for Hospital A and 18,832 records for Hospital B were retrieved. The mean age of patients (79 years Hospital A and 80 years Hospital B) was similar at both sites during the pre- and post-intervention periods, with approximately half being male. There was a noticeable increase in presentations at Hospital B with total presentations increasing from around 160 pre-GEDI to 180 post-GEDI (per week). There were no other large differences between the two sites or between the two time periods, see Table 2.

**Table 2:** Demographic and clinical characteristics of the sample in the two hospitals and two time periods

Characteristic	Hospital A Pre	Hospital A Post	Hospital B Pre	Hospital B Post
Presentations—number	9391	9349	8933	9899
Age – mean (sd)	79 [7]	79 (7)	80 (7)	80 (7)
Male sex – *n* (%)	4864 (52)	4922 (53)	4385 (49)	4733 (48)
Indigenous – *n* (%)	563 (6)	521 (6)	123 (1)	145 (1)
From RACF – *n* (%)	1017 (11)	1495 (16)	1250 (14)	1299 (13)
Presenting conditions – *n* (%)				
Cardiac	1753 (19)	1521 (16)	1554 (17)	2218 (22)
Trauma	999 (11)	895 (10)	1578 (18)	1646 (17)
Arrived by Ambulance – *n* (%)	5762 (61)	6453 (69)	6928 (78)	7527 (76)
ATS – *n* (%)				
Resus/ Emergent	2482 (26)	2398 (26)	2190 (25)	2776 (28)
Urgent	4614 (49)	4755 (51)	5166 (58)	5469 (55)
Less urgent/ Non-urgent	2295 (24)	2196 (23)	1577 (18)	1654 (17)
Seen by GEDI Team member – *n* (%)	0 (0)	3135 (33)	0 (0)	4151 (42)

Legend: *SD* Standard Deviation, *RACF* Residential Aged Care Facility, *ATS* Australasian Triage Scale

### Hospital A results

**Fig. 1:**
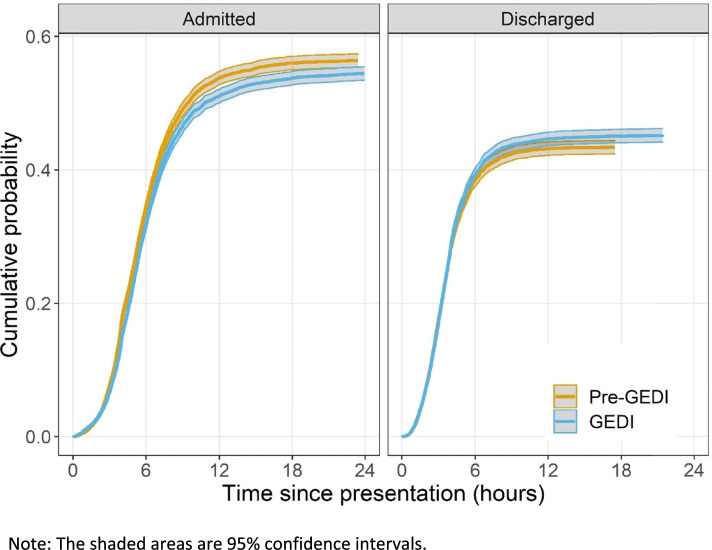
Cumulative probability of admission or discharge in the first 24 h after ED presentation during the pre- and post-GEDI period

There was an increased hazard (likelihood) of discharge, (shorter average time to discharge), during the post-GEDI period and if seen by a GEDI nurse, see Table 3. If a patient was seen by a GEDI nurse or seen during the post-GEDI period, there were increased numbers of faster discharges, with fewer admissions, visualized in Fig. 1. However, there was a significant increase in the time to admission during the post-GEDI period (Hazard ratio 0.88, CI 0.85,0.92) and if seen by a GEDI (Hazard ratio 0.76, CI 0.72,0.81). Table 3 highlights that the older the patient, the slower the discharge times (Hazard ratio 0.93, CI 0.92,0.95). If categorised in the MDC Trauma, there was also a significant increased risk of discharge. Presentations that were a lower ATS priority, such as minor trauma, rather than ATS priority of 1 or 2 (resuscitation or urgent) had an increased risk of admission. The post-GEDI period was associated with a reduction in the chance of re-presentation (Hazard ratio 0.88, CI 0.85,0.92).

**Table 3:** Survival models for Hospital A and Hospital B time to discharge during the post-GEDI period and if seen by a GEDI

	**HOSPITAL A**		**HOSPITAL B**		
**Variable**	**Hazard ratio**	**95% CI**	**Hazard ratio**	**95% CI**	
Period = Post-GEDI	1.08	1.03, 1.13	1.19	1.15, 1.24	
Seen by any GEDI = Yes	1.12	1.06, 1.18	1.14	1.09, 1.19	
Seen by GEDI nurse = Yes	N/A	N/A	1.14	1.08, 1.20	
Seen by GEDI Geriatrician = Yes	N/A	N/A	1.14	1.06, 1.23	
Seen by GEDI physiotherapist = Yes	N/A	N/A	1.18	1.10, 1.25	
Age (+ 5 years)	0.93	0.92, 0.95	0.96	0.94, 0.97	
Sex = Male	1.00	0.96, 1.05	1.01	0.97, 1.05	
Triage priority = Resuscitation/Emergent	0.32	0.30, 0.34	0.47	0.44, 0.50	
Triage priority = Urgent	0.49	0.47, 0.52	0.70	0.66, 0.73	
Mode of Arrival = Ambulance	0.57	0.54, 0.59	0.65	0.62, 0.68	
Trauma = Yes	1.57	1.47, 1.67	1.42	1.35, 1.49	
Cardiac = Yes	1.09	1.02, 1.17	1.11	1.05, 1.17	

Note: Hazard ratios above 1 indicate a greater risk (likelihood) of discharge and those below 1 a decreased risk (likelihood)

Results (Table 4) suggest that the risk of longer hospital stays (for those admitted) increased in the post-GEDI period overall but decreased for those seen by a GEDI nurse. The risk of in-hospital mortality decreased considerably in the post-GEDI period and if seen by a GEDI nurse, however the total number of deaths was small (pre-GEDI = 44, post-GEDI = 18).

**Table 4:** Secondary outcomes for GEDI model

**Secondary Outcome**	**Patients seen in post-GEDI period compared to the pre-GEDI period**		**Patients seen by GEDI compared to those not seen by GEDI in the post-GEDI period**	
	**HOSPITAL A**	**HOSPITAL B**	**HOSPITAL A**	**HOSPITAL B**
^a^Hospital LOS (days)	1.39 (1.21, 1.59)	-0.39 (-0.54, -0.23)	0.63 (0.41, 0.87)	-0.12 (-0.29, 0.05)
^b^Risk of in-hospital death	0.41 (0.24, 0.70)	0.66 (0.37, 1.16)	0.43 (0.15, 0.98)	0.84 (0.41, 1.59)
^c^Same cause ED representation	Not avail	0.96 (0.87,1.07)	Not avail	1.45 (1.29, 1.64)
^c^Any cause representation	0.89 (0.84, 0.95)	0.98 (0.92,1.05)	1.00 (0.92, 1.09)	1.60 (1.49, 1.73)
^c^Readmission any reason	1.07 (0.90, 1.28)	1.10 (0.88, 1.39)	1.21 (0.96, 1.53)	1.47 (1.14, 1.89)

Note: Cells show the mean estimates from multiple regression models and 95% confidence intervals in round brackets

^a^ days; ^b^ prevalence ratio; ^c^ hazard ratio

Overall, for all patients, the mean cost of ED presentation increased with increasing age of the patient (+ 5 years Age $15, (CI $12, $17;) and by an average of just $3 per presentation during the post-GEDI period (CI –$3, $9;) when compared to the pre-GEDI period. However, for patients who were admitted, while the cost increased the older the patient (+ 5 years Age $128, (CI $58, $198;)), the cost per admission decreased by an average of $88 per admission (CI –$270, $99;).

For patients seen by GEDI in the ED there was a small mean cost increase of $6 (CI –$2, $14), however, if admitted, there was a large mean cost reduction of $679 per stay (CI –$907, –$441) despite the hospital LoS being increased (0.63 days, (CI 0.41, 0.87)).

### Hospital B results

**Fig. 2:**
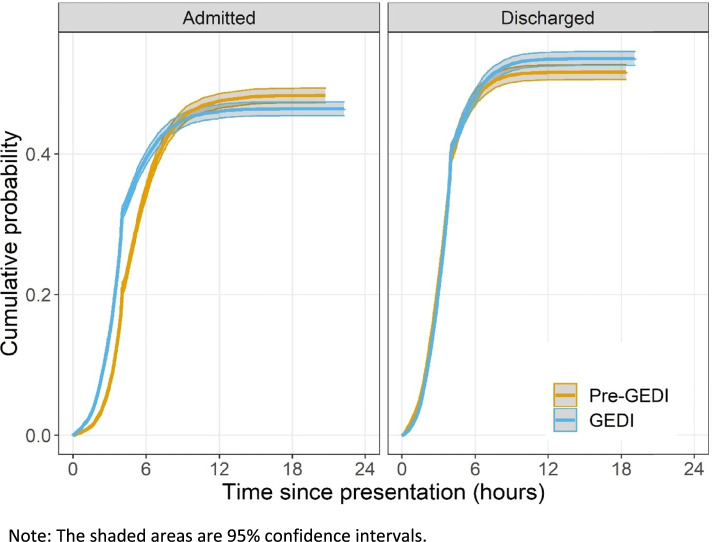
Cumulative probability of admission or discharge in the first 24 h after ED presentation during the pre- and post-GEDI period

There was a higher overall level of discharge and mirrored decrease in admission during the post-GEDI period. Admissions also tended to happen sooner in the post-GEDI period, see Fig. 2. The changes in the probability at four hours may be due to the National Emergency Access Target (NEAT) policy, that was in place at the time of data collection [24]. The post-GEDI period was associated with an increased hazard of time to discharge and admission (Table 3). Therefore, admissions and discharges occurred faster during the post-GEDI period compared to the pre-GEDI period, thus reflecting a decrease in risk of long ED LoS. All three GEDI clinicians (nurse, geriatrician and physiotherapist) and if ‘seen by any GEDI’ variables were associated with an increased risk of discharge, meaning faster times to discharge, (Table 3). The hazard ratios for time to admission by the GEDI nurses were well above 1 (1.17, CI 1.11,1.24) indicating a significant increased likelihood of shorter time to admission and shorter ED LoS. This was also seen for the GEDI Geriatrician (1.12, CI 1.03, 1.22), but the difference was smaller for the physiotherapist (1.07, CI 0.99, 1.15).

**Fig. 3:**
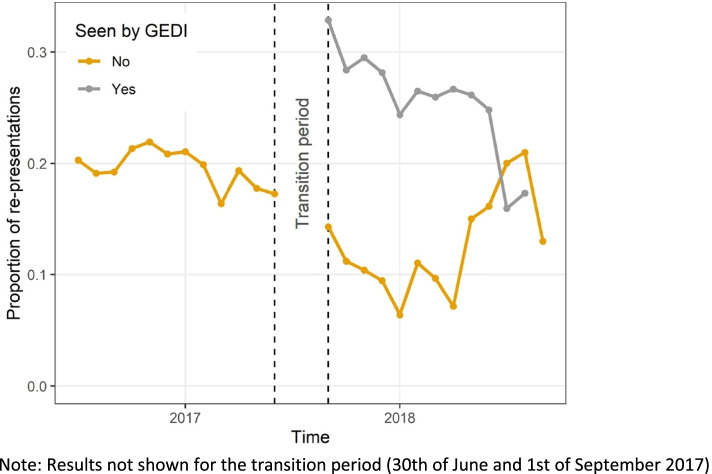
Monthly re-presentation probability over time

The post-GEDI period was associated with a shorter length of hospital stay for all patients admitted (not just those seen by any GEDI) (–0.39 days, CI –0.54, –0.23) with no change to representation for same (HR 0.96, CI 0.87, 1.97) or any-cause (HR 0.98, CI 0.92, 1.05), or risk of mortality. The risk of all-cause representation was significantly higher if seen by any GEDI, (Table 4). To further investigate this, we plotted the representation probability over time, (Fig. 3). The re-presentation times are generally consistently different between the two groups. However, the proportion of re-presentations drops for the group not seen by GEDI once the GEDI model starts.

**Fig. 4:**
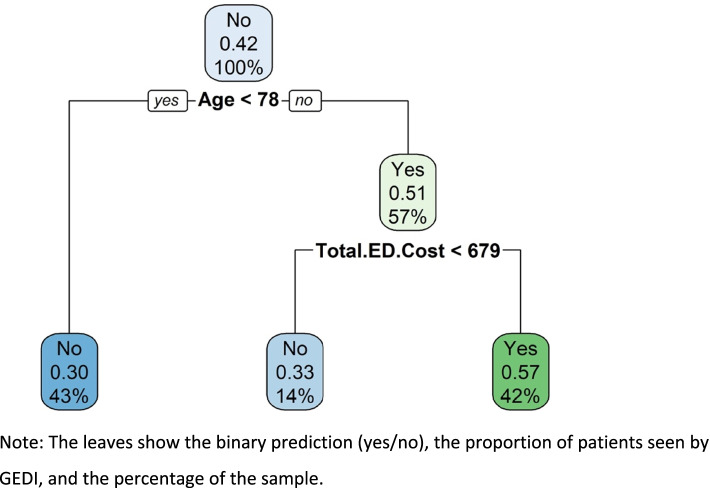
Classification tree estimating the probability of being seen by any GEDI

To further determine if GEDI consulted on more difficult cases we used a classification tree with a binary outcome of seen by GEDI or not (Fig. 4). The tree has two splits: age and cost. Patients aged under 78 had a 0.31 probability of seeing any GEDI clinician. Whereas patients over 78 whose total ED cost was over $671 had a 0.57 probability of seeing any GEDI clinician (which is 84% more likely).

Similar to Hospital A, the overall cost of ED presentation increased with increasing patient age (+ 5 years Age $7, CI $5, $9) and by an average of $41 per presentation during the post-GEDI period (CI $36, $47). However, for patients who were admitted, while the cost for the older patients increased (+ 5 years Age $175, CI $71, $281), the cost per admission during the post-GEDI period significantly decreased by an average of $532 per admission (CI –$771, -$278). Patients seen by any GEDI had an increased cost of $67 per patient (CI $60, $75) compared to patients not seen by any GEDI. The GEDI doctor was associated with an increase cost per admission of $235 (CI $38, $452).

## Discussion

In this study we aimed to evaluate the effectiveness of the previously described GEDI model after its implementation into two different EDs. The results indicate that the GEDI model can be successfully translated. Both sites showed that the implementation of the GEDI model increased the likelihood of discharge, decreased ED LoS, decreased hospital costs for those admitted, with an associated reduction in risk of mortality, for adults aged 70 years and over. Of note, in Hospital A the cost of the admission in the post-GEDI period if seen by a GEDI was less despite having a longer hospital LoS. This may be explained by the GEDI team’s input guiding the inpatient team’s decisions around the appropriateness of costly interventions and investigations. In addition, there was an overall reduction in hospital LoS at Hospital B during the post-GEDI period compared to the pre-GEDI period regardless of being seen by GEDI or not. Within the Hospital B local government area, there was a 11% increase in population > 70 years in the during the pre- and post-GEDI period [25] likely contributing to the increased presentations at Hospital B. This may explain the decrease in hospital LoS in all patients in the post-GEDI period due to increased hospital bed pressures at Hospital B. Given GEDI staff prioritised older adults who screened with increased physical and cognitive dependency and GEDI’s role is to identify more issues earlier, it is not surprising that patients seen by GEDI had a longer hospital LOS, if admitted, compared with those not seen by GEDI at both hospitals. Similarly, the finding that the proportion of re-presentations drops for the group not seen by GEDI once the GEDI model starts implies that the GEDI team correctly targeted patients with greater cognitive and physical dependencies.

Implementation issues are prevalent in the field of geriatric medicine due to the complexity of care for many older adults. Existing interventions require adaptation across both healthcare disciplines and settings [26]. Similarly, Dresden et al. [27] observed a difference in outcomes across sites despite implementing the same model of care. One site observed no difference in admission rates after assessment by a geriatric care nurse, despite a consistent, robust implementation framework [27]. Interventions cannot always be implemented with a duplicate program model as differing environments often require program adaptations. While some research findings suggest successful interventions are dependent on adaptations to improve the fit of the model across sites [28], it is well recognised that model fidelity is integral to successful implementation [29]. Motivation, ownership and engagement of clinicians is a key component of retaining fidelity, however, these factors can be difficult to maintain throughout the stages of implementation across multiple sites [30, 31]. There is limited research examining how clinician engagement results in challenges to implementation within the ED. We attribute the variation in outcomes to differences in motivation and engagement within the multi-disciplinary team rather than model fidelity due to validated frameworks being use in our implementation process. However, further research is needed to examine this observation.

Both sites employed dedicated GEDI nurses into their EDs, however, Hospital B also employed a GEDI doctor and physiotherapist. Overall, in Hospital A, the introduction of GEDI nurses reduced time spent in ED and increased the likelihood of safe discharge for adults aged over 70 years whilst reducing ED and hospital costs. However, in Hospital B all three GEDI clinicians accomplished a decrease in overall risk of admission and a decrease in time to discharge and admission. When reviewing the GEDI doctor in isolation, there was an associated increased hospital LoS and significantly increased total hospital cost. This is unsurprising given the likely reverse causality of doctors being involved in the more complex and sicker patients with an associated increased staffing cost. This observation was also reflected in the sub analysis of the representation data for all GEDI clinicians at hospital B. In future studies, comparison of patient level data and associated healthcare costs/outcomes, to determine the effectiveness of the GEDI doctor in the model and to ascertain the complexity of the patients being seen by the GEDI team, would be of benefit. Risk scores [32, 33] could be used to stratify patients for GEDI doctor review and examined in future studies.

Implementation of interventions into complex adaptive systems are not without their challenges. Despite comprehensive frameworks described in the literature, the availability of the core components in these frameworks may not be obtainable. This may explain the differences in the outcomes measured between the pilot site where GEDI was created and the implementation sites in this study. The motivation of facilitators is recognised as a key concept for successful implementation in the i-PARIHS framework. Hasson et al. [28] observed that positive staff experiences, in similar successful projects, had a positive effect on motivation and responsiveness of the new project staff which in turn influenced facilitation strategies and project implementation. While there were also contextual factors that hindered facilitators, the impact of these factors on fidelity were adjusted by other moderating factors such as staff motivation and the responsiveness of clinicians [28]. Thus, the implementation of the model across sites where staff and clinicians have limited experience in geriatric models of care, would likely have a multifactorial effect on model fidelity.

Having a geriatric focussed/embedded team in the ED may have had a global impact on the way ED staff approached the care of the older adult in their respective EDs. In comparison with the pre-GEDI period, both EDs saw an improvement in the likelihood of discharge for all older adult patients after implementation of the GEDI model, whether the patient was seen by the GEDI team or not. A contamination effect may have occurred due to the intervention principles being adopted by the other ED staff when the intervention or GEDI team member was not available. This diffusion of improved outcomes may have diluted the observed differences between the older adults’ outcomes if seen or not seen by a GEDI [34, 35]. If GEDI’s were directly involved in the care of the patient, we saw a significant reduction in hospital length of stay, for those admitted. This suggests the targeted approach that GEDI adopt mean they focus on the cases that can be more easily discharged and those that require hospitalisation. This approach means significant reductions in hospital costs associated with ED presentation by older adults.

### Limitation of the implementation

Patient level outcomes such as functional decline and quality of life were not investigated in these models. While prospective quality of life measurements may be biased [36], other outcome measures including patient experience would strengthen the findings. Randomisation is considered the most robust method of preventing selection bias, however the practical and ethical objections to evaluating an intervention that has previously shown benefit often guides researchers to quasi experimental designs such as this one. Future implementation studies may consider utilising a multisite stepped wedge design. This allows the intervention to be introduced to groups of clusters over time and decreases the risk of attempting implementation at multiple sites all at once. Especially if facilitation of the implementation is at risk by doing so. Furthermore, the researchers can record and analyse changes that may occur in the hospitals over time that may have affected the study outcomes.

This pre-post design has looked at the outcomes of the implementation of different versions of the GEDI model into two separate EDs. The process by which these implementations occurred, the challenges faced, and the qualitative outcomes are beyond the scope of this article and are submitted for publication separately. As the population of Australia ages, EDs are facing unprecedented levels of demand for quality healthcare. The rapid translation of research and implementation of novel models of care that focus on improving the care of this vulnerable population in ED is going to be critical. These models aim to reduce inappropriate hospitalisation whilst minimising harm from hospital acquired complications.

## Conclusion

Older adults in the ED are at higher risk of negative outcomes and current ED practice often focusses on the management of their presenting complaint without taking due account of their dependence and/or the presence of geriatric syndromes. Different approaches have been described in the literature to combat this issue including dedicated geriatric EDs, separate frailty units and embedded clinical expertise within the ED. The GEDI model is a successful example of an embedded ED model of care targeting the highly dependent older adult in ED. This model was implemented in to two EDs to ascertain its translatability.

Translating research and implementing models into complex adaptive healthcare systems is challenging. GEDI although seemingly successful in one healthcare system, can be seen to have more subtle outcomes when translated into others. The results highlight the benefit of a program embedded in the ED that focusses on a vulnerable population. This was seen through the comparison of the pre-GEDI versus post-GEDI periods. Interventions must change, adapt, and evolve in new sites because the culture and context of the local environment is important to ensure innovation adoption. However, there must also be an understanding that despite some compromises being made the principles of the intervention and its associated key features must be retained.

## Supplementary Information

**Additional file 1. **STROBE Statement—Checklist of items that should be included in reports of cohort studies 

## Data Availability

Data are available on request due to privacy or other restrictions: The data that support the study findings are available on request from the corresponding author (MW/AC). The data are not publicly available due to ethics restrictions and them containing information that could compromise research participant privacy/consent.

## Abbreviations

GEDI: Geriatric Emergency Department Intervention
ED: Emergency Department
i-PARIHS: Integrated Promoting Action on Research Implementation in Health Services
PARIHS: Promoting Action on Research Implementation in Health Services
LoS: Length of stay
ATS: Australasian Triage Score
Sd: Standard Deviation
RACF: Residential Aged Care Facility
CI: Confidence Interval
NEAT: National Emergency Access Target

## References

[1] Ellis G, Whitehead MA, Robinson D, O’Neill D, Langhorne P. Comprehensive geriatric assessment for older adults admitted to hospital: meta-analysis of randomised controlled trials. BMJ. 2011;343: d6553. 10.1136/bmj.d6553]. 10.1136/bmj.d6553 PMC3203013 22034146

[2] Pilotto A, Cella A, Pilotto A, Daragjati J, Veronese N, Musacchio C, et al. Three Decades of Comprehensive Geriatric Assessment: Evidence Coming From Different Healthcare Settings and Specific Clinical Conditions. J Am Med Dir Assoc. 2017;18(2):192.e1-.e11. 10.1016/j.jamda.2016.11.004. 10.1016/j.jamda.2016.11.004 28049616

[3] Aldeen A, Courtney D, Lindquist L, Dresden S, Gravenor S (2014). Geriatric Emergency Department Innovations: Preliminary Data for the Geriatric Nurse Liaison Model. J Am Geriatr Soc.

[4] Conroy S, Ansari K, Williams M, Laithwaite E, Teasdale B, Dawson J, et al. A controlled evaluation of comprehensive geriatric assessment in the emergency department: the “Emergency Frailty Unit.” Age Ageing. 2014;43(1):109–14. 10.1093/ageing/aft087]. 10.1093/ageing/aft087 PMC3861335 23880143

[5] Wallis M, Marsden E, Taylor A, Craswell A, Broadbent M, Barnett A, et al. The Geriatric Emergency Department Intervention model of care: a pragmatic trial. BMC Geriatr. 2018;18(1):297. 10.1186/s12877-018-0992-z. 10.1186/s12877-018-0992-z PMC6276263 30509204

[6] Graf CE, Zekry D, Giannelli S, Michel JP, Chevalley T (2011). Efficiency and applicability of comprehensive geriatric assessment in the emergency department: a systematic review. Aging Clin Exp Res.

[7] Marsden E, Taylor A, Wallis M, Craswell A, Broadbent M, Barnett A (2019). Effect of the Geriatric Emergency Department Intervention on outcomes of care for residents of aged care facilities: A non-randomised trial. Emerg Med Australas.

[8] Marsden E, Taylor A, Wallis M, Craswell A, Broadbent M, Johnston-Devin C (2021). A structure and process evaluation of the Geriatric Emergency Department Intervention model. Australasian Emergency Care.

[9] Walker K, Tan SI, Fatovich D, Watkins G, Stephenson M, Ting J (2020). Research capacity of Australian and New Zealand emergency medicine departments. Int J Emerg Med.

[10] Eccles, MP, Mittman BS. Welcome to Implementation Science. Implementation Sci. 2006;1:1. 10.1186/1748-5908-1-1.

[11] Dearing JW, Cox JG (2018). Diffusion Of Innovations Theory, Principles. And Practice Health Affairs.

[12] Birken SA, Powell BJ, Shea CM, Haines ER, Alexis Kirk M, Leeman J (2017). Criteria for selecting implementation science theories and frameworks: results from an international survey. Implement Sci.

[13] Rogers EM. Diffusion of innovations. New York: The Free Press; 2010. ISBN:9781451602470.

[14] Harvey G, Kitson A (2016). PARIHS revisited: from heuristic to integrated framework for the successful implementation of knowledge into practice. Implement Sci.

[15] Michie S, Johnston M, Abraham C, Lawton R, Parker D, Walker Aea (2005). Making psychological theory useful for implementing evidence based practice: a consensus approach. Qual Saf Health Care.

[16] Damschroder L, Aron DC, Keith R, Kirsh SR, Alexander J, Lowery J (2009). Fostering implementation of health services research findings into practice: a consolidated framework for advancing implementation science. Implement Sci.

[17] Kitson A, Harvey G, McCormack B (1998). Enabling the implementation of evidence based practice: a conceptual framework. Qual Health Care.

[18] Marsden E, Taylor A, Wallis M, Craswell A, Bannink N, Broadbent M, et al. Geriatric Emergency Department Intervention (GEDI) toolkit Brisbane: Clinical Excellence Queensland; 2018. https://clinicalexcellence.qld.gov.au/resources/gedi-toolkit

[19] interRAI (2022) Screeners: Emergency Department Screener (ED) and Contact Assessment (ED-CA) https://interrai.org/instrument-category/screeners/

[20] Chen L-J, Chen C-Y, Lue B-H (2014). Prevalence and Associated Factors of Frailty Among Elderly People in Taiwan. Int J Gerontol.

[21] Baker R, Camosso-Stefinovic J, Gillies C, Shaw EJ, Cheater F, Flottorp S, et al. Tailored interventions to address determinants of practice. The Cochrane database of systematic reviews. 2015;2015(4):Cd005470. 10.1002/14651858.CD005470.pub3]. 10.1002/14651858.CD005470.pub3 PMC7271646 25923419

[22] Queensland CE. ICD-10 code mapping. Brisbane: Queensland Health; 2015. https://clinicalexcellence.qld.gov.au/sites/default/files/docs/gedi/code-mapping.pdf

[23] Australasian College for Emergency Medicine. Guidelines for the Implementation of the Australasian Triage Scale (G24). 2013. https://acem.org.au/getmedia/51dc74f7-9ff0-42ce-872a-0437f3db640a/G24_04_Guidelines_on_Implementation_of_ATS_Jul-16.aspx

[24] Sullivan C, Staib A, Khanna S, Good NM, Boyle J, Cattell R (2016). The National Emergency Access Target (NEAT) and the 4-hour rule: time to review the target. Med J Aust.

[25] Australian Bureau of Statistics. Region summary: Ipswich (C) 2018. https://dbr.abs.gov.au/region.html?lyr=lga&rgn=33960

[26] Reckrey JM, Gazarian P, Reuben DB, Latham NK, McMahon SK, Siu AL (2021). Barriers to implementation of STRIDE, a national study to prevent fall-related injuries. J Am Geriatr Soc.

[27] Dresden S, Hwang U, Garrido M, Sze J, Kang R, Vargas-Torres C (2020). Geriatric Emergency Department Innovations: The impact of transitional care nurses on 30-day readmissions for older adults. Acad Emerg Med Off J Soc Acad Emerg Med.

[28] Hasson H, Blomberg S, Dunér A. Fidelity and moderating factors in complex interventions: a case study of a continuum of care program for frail elderly people in health and social care. Implementation science 2012;7(23). 10.1186/1748-5908-7-23. 10.1186/1748-5908-7-23 PMC3342887 22436121

[29] McGee D, Lorencatto F, Matvienko-Sikar K, Toomey E (2018). Surveying knowledge, practice and attitudes towards intervention fidelity within trials of complex healthcare interventions. Trials.

[30] Geerligs L, NM R, HL S, P. B. Hospital-based interventions: a systematic review of staff-reported barriers and facilitators to implementation processes. Implementation science : IS. 2018;13(1):36. 10.1186/s13012-018-0726-9 10.1186/s13012-018-0726-9 PMC5824580 29475440

[31] Frykman M, Hasson H, Athlin ÅM, von Thiele SU (2014). Functions of behavior change interventions when implementing multi-professional teamwork at an emergency department: a comparative case study. BMC Health Serv Res.

[32] Kaeppeli T, Rueegg M, Dreher-Hummel T, Brabrand M, Kabell-Nissen S, Carpenter CR (2020). Validation of the Clinical Frailty Scale for Prediction of Thirty-Day Mortality in the Emergency Department. Ann Emerg Med.

[33] Wallis SJ, Wall J, Biram RWS, Romero-Ortuno R. Association of the clinical frailty scale with hospital outcomes. QJM. 2015;108(12):943–9. 10.1093/qjmed/hcv066. 10.1093/qjmed/hcv066 25778109

[34] Dryden-Palmer KD, Parshuram CS, Berta WB (2020). Context, complexity and process in the implementation of evidence-based innovation: a realist informed review. BMC Health Serv Res.

[35] Wisdom JP, Chor KH, Hoagwood KE, Horwitz SM (2014). Innovation adoption: a review of theories and constructs. Adm Policy Ment Health.

[36] Blome C, Augustin M. Measuring change in quality of life: bias in prospective and retrospective evaluation. Value in health : the journal of the International Society for Pharmacoeconomics and Outcomes Research. 2015;18(1):110–5. 10.1016/j.jval.2014.10.007]. 10.1016/j.jval.2014.10.007 25595241

